# Case Report: A Severe SARS-CoV-2 Infection in a Teenager With Angelman Syndrome

**DOI:** 10.3389/fmed.2021.629112

**Published:** 2021-03-12

**Authors:** Alessandra G. D. Lopes, Camila S. H. Celestino, Tiago T. A. Barros, Aline G. Fevereiro, Debora H. Gejer, Fernando M. F. Oliveira, Jamile M. Brasil, Rosely M. Bossolan, Gabriela C. C. Pinto, Ana C. E. Z. Santos, Luis A. Divan, Ingrid A. B. Alves, Danielle B. L. Oliveira, Rafael R. G. Machado, Luciano M. Thomazelli, Meire I. Hiyane, Leonília Brelaz-Abreu, Elayne Bragança-Jardim, Letícia B. S. Heinen, Anna C. M. Barrientos, Luciana B. Mau, Niels O. S. Camara, Daniela F. Bueno, Mariane T. Amano

**Affiliations:** ^1^Hospital Municipal Infantil Menino Jesus, São Paulo, Brazil; ^2^Hospital Sírio-Libanês, São Paulo, Brazil; ^3^Departamento de Microbiologia, Instituto de Ciências Biomédicas, Universidade de São Paulo, São Paulo, Brazil; ^4^Hospital Israelita Albert Einstein, São Paulo, Brazil; ^5^Departamento de Imunologia, Instituto de Ciências Biomédicas, Universidade de São Paulo, São Paulo, Brazil

**Keywords:** COVID19, SARS-CoV-2, Angelman syndrome, neutralizing antibodies, intensive care unit, inflammation, immune response

## Abstract

Teenagers generally present mild to no symptoms of severe acute respiratory syndrome coronavirus 2 (SARS-CoV-2) infection. In the present report, we present the case of a 14-year-old boy with Angelman syndrome (AS) who presented with severe COVID-19 symptoms. He spent 20 days in the ICU with elevated inflammatory biomarkers (C-reactive protein and D-dimer) and increased peaks of neutrophil-to-lymphocyte ratio, which is uncommon for teenagers diagnosed with COVID-19. Although he showed physiological instability, he was able to produce neutralizing antibodies, suggesting a functional immune response. The literature concerning the immune response to infections in patients with AS is still poor, and to our knowledge, this was the first report of a patient with AS diagnosed with COVID-19. As such, the present study may alert other patients with AS or other rare diseases that they lack a competent immune response and could suffer severe consequences of SARS-CoV-2 infection.

## Introduction

Coronaviruses are enveloped, non-segmented, positive-sense RNA viruses belonging to the family Coronaviridae. The latest human pathogenic coronavirus to be identified was SARS-CoV-2, which causes COVID-19 (1). It was first reported in December 2019 in Wuhan, China, and declared a pandemic by the World Health Organization (WHO) in March 2020. It continued to spread across the globe, alternating hotspots during the last months (2). The number of confirmed pediatric cases is much lower than that of adult cases, and the severity and mortality rates are even lower (3). However, at all ages, patients with hypertension, diabetes, obesity, or chronic lung disease are more prone to severe disease (2). In the present report, we describe the case of a 14-year-old boy with asthma and Angelman syndrome (AS) who developed a life-threatening manifestation of COVID-19 due to severe respiratory distress syndrome. Although allergy does not appear to be a predisposing factor for COVID-19 (4), it is well-documented that children with neurological disorders are more prone to develop severe clinical course in respiratory tract infections (5).

## Case Description

A 14-year-old boy with asthma and AS visited the emergency department of Hospital Municipal Infantil Menino Jesus, São Paulo, Brazil in April 2020; he had a history of fever episodes of 38°C, respiratory distress, and perioral cyanosis. At home, he had performed inhalation with salbutamol and ipratropium, but developed worsening wheezing. When he arrived at our center, he was hypothermic (34.8°C), tachycardic, tachydyspneic, and had an O_2_ saturation of 77% on peripheral oximetry. His mother also had flu-like symptoms and complained of anosmia. At that time, Brazil was in quarantine and hospitals were implementing extensive measures to prevent COVID-19, providing personal protective equipment to hospital care team members, patients, and their families. In addition, our center was executing a specific COVID-19 protocol to isolate patients who met the following criteria: fever, cough, runny nose, shortness of breath, difficulty breathing with hospital admission needed, or underlying uncontrolled medical condition. With the hypothesis of severe acute respiratory syndrome due to infection by SARS-CoV-2, the patient was admitted to the hospital and subjected to a viral panel (6), which included adenovirus (AdV), respiratory syncytial virus (RSV-A, -B), parainfluenza virus (PIV-1,−2,−3,−4), influenza virus (Flu-A, -B), human metapneumovirus (hMPV), seasonal coronavirus (CoV-OC43, -HKU1, -NL63,−229E), enterovirus (EV), rhinovirus (RV), and SARS-CoV-2 (7).

**Figure 1:**
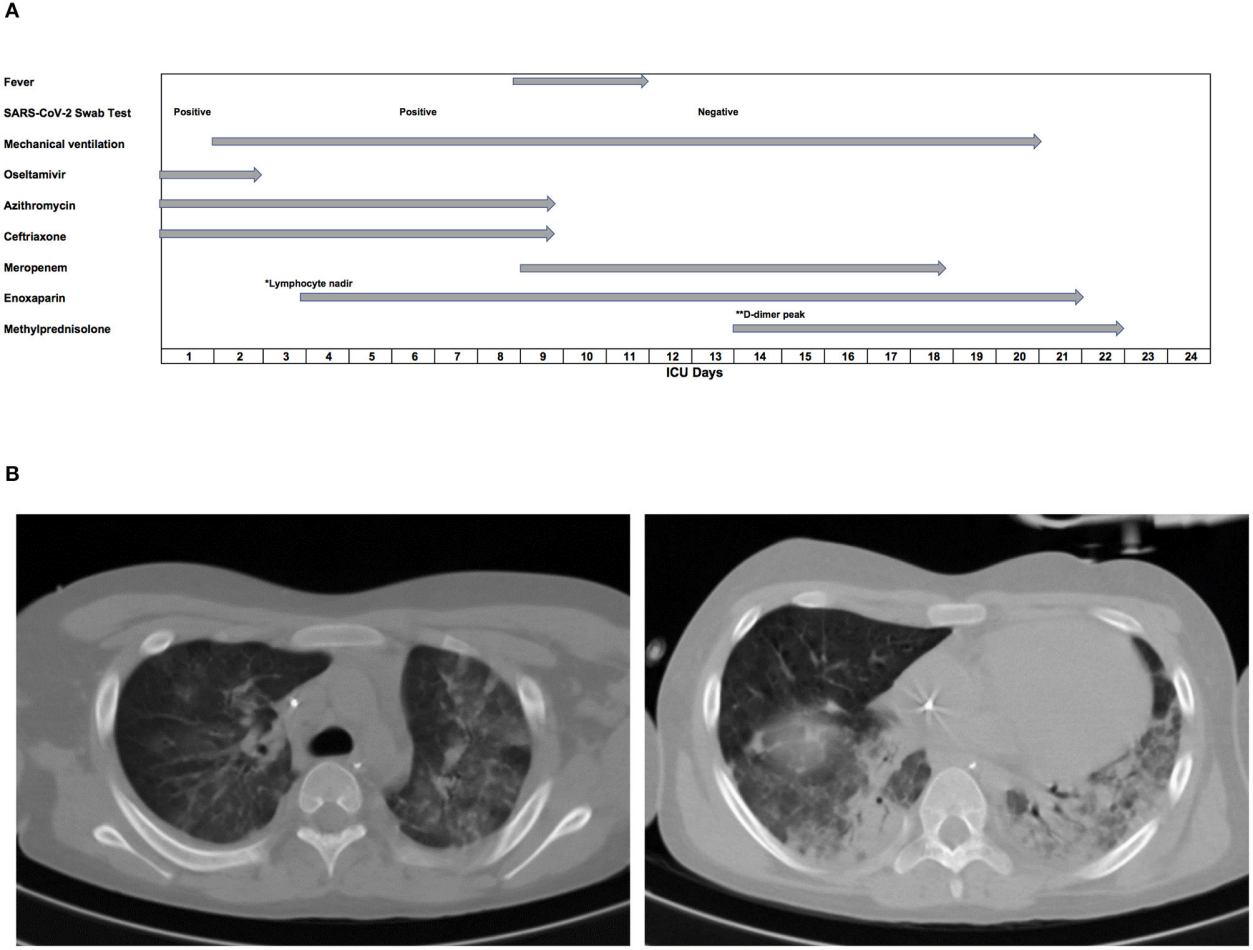
Clinical parameters evolution. **(A)** Timeline of symptoms and treatment during ICU stay. **(B)** Chest tomography of the fourth day of hospitalization with considerable pulmonary consolidation in the left lower lobe and in a smaller part of the right, with diffuse ground-glass densification in the unconsolidated pulmonary portions. ICU, intensive care unit. *Lymphocyte = 1.21 × 10^9^/L; **D-dimer = 2,023.5 (ng/mL).

**Figure 2:**
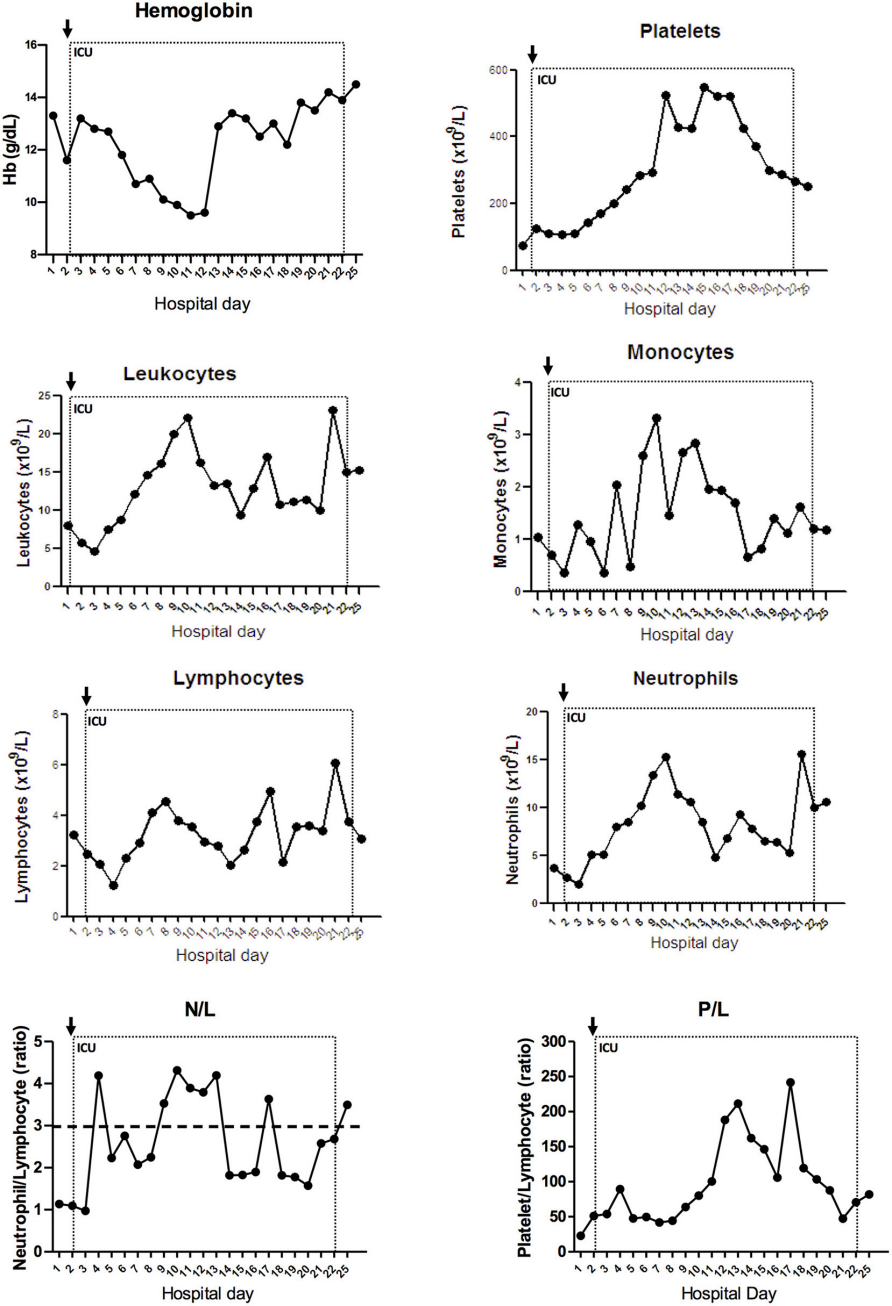
Evolution of blood count markers during hospitalization. Hemogram was evaluated along the hospital stay. Hemoglobin, platelet, total leukocytes, monocyte, lymphocyte, neutrophil count, and the ratios neutrophil-to-lymphocyte (N/L) and platelet-to-lymphocyte (P/L) are represented with connected scatterplot. ICU, intensive care unit. Dotted line represents the ICU stay and the arrow indicates ICU admission. Dashed line indicates N/L threshold with ratio over three associated with severe illness in adults (7).

**Figure 3:**
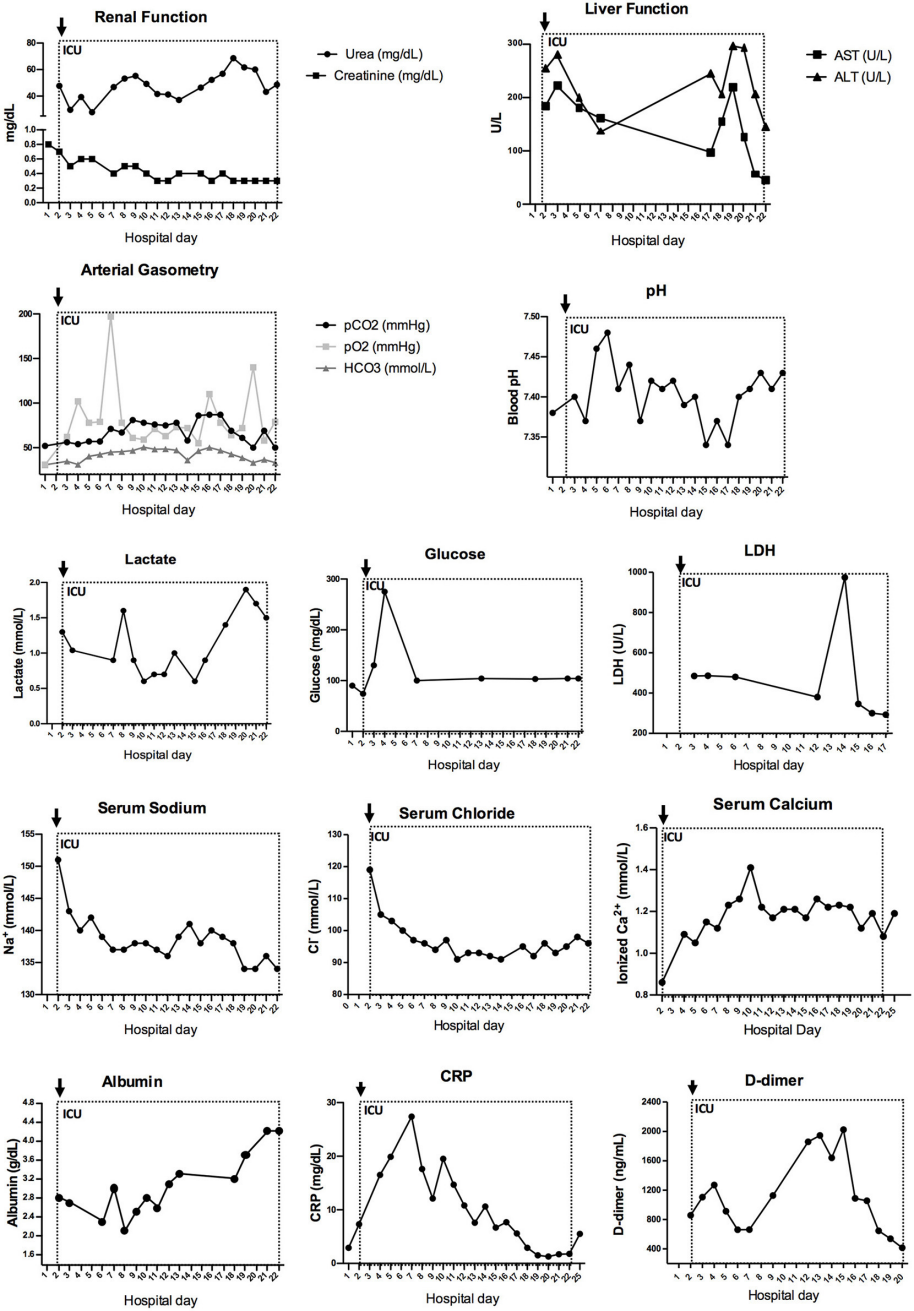
Increased inflammatory profile is observed in arterial gasometry and biochemical markers during hospitalization. Urea and creatinine are combined to represent renal function. AST and ALT are combined to resepresent liver function. Arterial gasometry is shown in pCO2, pO2, and HCO3 combined graph and pH measures. Together with lactate, glucose, LDH, serum sodium, serum chloride, serum calcium, albumin, CRP and D-dimer, these connected scatterplot show the phisiological evolution of the patient during ICU stay. AST, aspartate transaminase; ALT, alanine aminotransferase; pCO2, partial pressure of carbon dioxide; pO2, partial pressure of oxygen; HCO3, serum bicarbonate; CRP, C-reactive protein; ICU, intensive care unit. Dotted line represents the ICU stay and the arrow indicates ICU admission.

Initial chest X-ray showed multiple bilateral consolidation foci with a cottony aspect. The patient received supplemental oxygen therapy, volume expansion with saline solution, oseltamivir, and antibiotic therapy with azithromycin and ceftriaxone. He evolved on his second day of hospitalization, with worsening of his breathing pattern, and was transferred to the intensive care unit (ICU). Mechanical ventilation was initiated on the following day (Figure 1A). At this time, the patient had mild anemia (hemoglobin: 11.6 g/dL, normal reference [NR]: 13.0–17.0 g/dL) and thrombocytopenia (platelet count: 125 × 10^9^/L, NR: 150–400 × 10^9^/L), with normal leukocytes (5.83 × 10^9^/L, NR: 4.5–11 × 10^9^/L), and monocytes (3.27 × 10^9^/L, NR: 1–4 × 109/L). Although lymphopenia is commonly seen in patients with severe COVID-19, lymphocyte count was normal in the patient (2.45 × 10^9^/L, NR 1–3 × 10^9^/L), as were platelet to lymphocyte ratio and neutrophil count. However, the neutrophil-to-lymphocyte (N/L) ratio was elevated during the patient's stay in ICU (Figure 2). Although renal function was not affected, as indicated by creatinine and urea levels, increased liver enzymes were observed [aspartate aminotransferase (AST): 184 U/L, alanine aminotransferase (ALT): 253 U/L; NR 15–40 U/L], as were hypoalbuminemia (2.8 g/dL, NR: 3.5–5 g/dL), and hypernatremia (151 mmol/L, NR: 137–145 mmol/L). The patient's serum chloride levels were also elevated (119 mmol/L, NR: 98–107 mmol/L), while his ionized calcium levels were decreased (0.86 mmol/L, NR: 1.12–1.32 mmol/L). Other electrolytes presented normal levels in the serum, as follows: lactate (1.3 mmol/L, NR: 0.5–1.6 mmol/L), potassium (4.4 mmol/L), phosphate (4.1 mg/dL), and magnesium (1.8 mg/dL). Elevated ferritin levels (483 ng/mL, NR: 7–140 ng/mL) were found in the blood on the following day, indicating liver damage. D-dimer and C-reactive protein (CRP) were somewhat elevated (D-dimer: 858.1 ng/mL, NR <500 ng/mL; CRP: 7.3 mg/dL, NR <1 mg/dL), and lactate dehydrogenase (LDH) showed increased levels (636 U/L, NR: 120–246 U/L) (Figure 3). These three biomarkers are positively correlated with the severity of COVID-19 (8).

Blood culture was negative, confirming no bacterial infection. On day 2, the result of the viral panel confirmed infection with SARS-CoV-2, with a swab qPCR viral load of 5.9 × 10^6^ RNA copies/mL and a cycle threshold (Ct) 24.72 (9). The patient was negative for other viruses (Figure 1A). Thus, oseltamivir was suspended and a viral panel from the patient's mother was collected, which later identified the same virus. The patient remained on mechanical ventilation for 19 days.

On day 4 of hospitalization, a chest CT scan was performed (Figure 1B), demonstrating extensive pulmonary consolidation in the left lower lobe and in a limited part of the right lobe. Diffuse ground-glass densification was observed in the unconsolidated pulmonary portions. The scan also showed moderate pleural effusion on the left side. On day 5 of hospitalization, echocardiography showed a hyper-refringent image in the inferior vena cava, suggestive of a thrombus measuring 15.5 × 8.7 mm. Enoxaparin was started at an anticoagulant dose until the lesion resolved 18 days later. A progressive increase in D-dimer was observed, with the initial level of 858.1 ng/mL reaching a peak of 2023.5 ng/mL on day 15 of hospitalization (Figure 3). On day 6 of hospitalization, the SARS-CoV-2 test was repeated and demonstrated a decrease in viral load to 3.5 × 10^4^ copies/mL, with a Ct value of 32.20. On day 10 of ceftriaxone and azithromycin, the patient started to show feverish peaks and worsening respiratory parameters. Laboratory tests showed leukocytosis (22.2 × 10^9^/L), with a left shift. Antibiotic therapy was staggered with meropenem because the patient was suspected to have pneumonia associated with mechanical ventilation. He evolved with improvement of the fever on day 3 of meropenem and completed a total of 10 days of antibiotic therapy. On day 14 of hospitalization, the viral panel was repeated, with a negative result. On day 15 of hospitalization, corticosteroid therapy with methylprednisolone was started, prompted by his pulmonary condition and personal history of asthma. We intended to carry out 5 days of this treatment, but it was extended for another 4 days as the patient started to show urticaria. He subsequently showed progressive improvement in respiratory parameters and was extubated on day 21 of hospitalization. The criteria for hospital discharge were clinical resolution of symptoms, stabilization of underlying diseases, and negative PCR test for SARS-CoV-2. The boy was discharged from hospital negative for SARS-CoV-2 and in good general condition and controlled asthma, without the need for home oxygen therapy. In total, he had been hospitalized for 36 days, with 20 days in intensive care.

After 80 days, neutralizing antibodies (nAbs) was measured in the patient's plasma; the neutralization end point titer was 640 (high titer), while the expected negative control titer was <20. The methodology for quantification of nAbs was previously described by Wendel et al. (10). Cytokines were also measured at this time using an 8-Plex Human Cytokine Kit (BioRad); lower levels of inflammatory cytokines (IL-8 and TNFa) were detected in the patient's plasma (Table 1) than control (six children without AS that had COVID-19).

**Table 1:** Long-term plasma cytokines.

**Cytokines**	**Patient**	**Control**
**(pg/mL)**		
IL-2	0	0.33 (0.00–0.82)
IL-4	0.11	0.12 (0.01–0.18)
IL-6	0	0.44 (0–2.29)
IL-8	3.37	8.58 (3.62–18.07)
IL-10	0	2.21 (0–12.06)
IFNg	0.35	0.47 (0.30–1.16)
TNFa	14.55	36.00 (16.1–60.57)

*Parentheses in Control group are minimum and maximum value*.

## Discussion

AS is a rare neurogenetic disorder characterized by microcephaly, severe intellectual deficit, speech impairment, epilepsy, EEG abnormalities, ataxic movements, tongue protrusion, paroxysms of laughter, abnormal sleep patterns, and hyperactivity (11). It has a prevalence of between 1/10,000 and 1/20,000 individuals (12) and is considered a syndromic form of autism spectrum disorder (13). AS results from loss of function of the imprinted ubiquitin-protein ligase E3A (*UBE3A*) gene on chromosome 15q11.2-q13 (11, 14). Although life expectancy appears to be normal, severe complications can occur due to some of the syndrome's symptoms, such as seizures and aspiration pneumonia (12).

To our knowledge, no reports to date have described COVID-19 in patients with AS. Several studies have reported that the signs and symptoms of COVID-19 in children are similar to those in adults, but milder (15). In a recent report, abnormalities in chest CT images were detected among all patients upon admission (16). Typical images included bilateral multiple lobular and subsegmental areas of consolidation, as well as bilateral ground-glass opacity (16). These findings corroborate those found in our patient. In the same study, the average time between hospitalization and admission to the ICU was 3 days (16), which was about the time at which our patient showed symptom worsening and required mechanical ventilation.

Studies have shown lymphopenia is the most common hemogram finding in adults with COVID-19, occurring in as many as 70.3–83% of hospitalized patients (15), while only being observed in 9.8% of cases (15). This is consistent with the findings in our patient, who showed neither leukopenia nor lymphopenia upon admission to the ICU. In contrast, an N/L ratio > 3.13 has been reported as a risk factor for developing severe illness in adults (8), and our patient presented peaks of N/L ratio over 3. In children, there is likely a negative correlation between LDH levels and severity (17). The literature is still sparse regarding the immune system of patients with AS, although the close relationship with autism may suggest that these patients have a dysfunctional immune response (18). In adulthood, males are prone to infectious illnesses (19). Moreover, one report described a 5-year old boy with AS who had recurrent respiratory infections, hyperactivity, and sleep disturbances; he died of upper airway obstruction due to infectious mononucleosis (20). This case supports the idea that the immune response is compromised in patients with AS. More recently, UBE3A was found to regulate interferon regulatory factor (IRF); lack of UBE3A leads to AS, and interferon cytokines are essential to the anti-viral immune response, so this may intimate a mechanism for the impaired immune response in patients with AS (21). In the present study, we demonstrated that the humoral immune response had been activated, since the level of nAbs for SARS-CoV-2 was detected in the patient's plasma 80 days after discharge. Curiously, the levels of inflammatory cytokines were lower than in the control. Further studies are required to determine whether this was a consequence of the infection or was related to the patient's AS.

The overall levels of acute phase reactants in COVID-19 correlates with disease severity and death. According to a recent study involving 140 hospitalized patients, higher levels of D-dimer and CRP were associated with severe disease (2). In the present case, the patient presented with a slight elevation in CRP and D-dimer levels upon admission to the ICU; the levels progressively increased subsequently, reaching a peak of more than four times the normal value on day 15 of hospitalization, probably because of the excessive inflammatory process accompanied by SARS-CoV-2 infection (22–24). D-dimer is also a marker of coagulation problems, which are also a feature of severe COVID-19 (23). As reported, on the fifth day of hospitalization, echocardiogram detected an image suggestive of a thrombus in the inferior vena cava, and anticoagulant treatment was initiated.

Neurological diseases can increase the risk of lower respiratory tract infection (5). In the present case, the patient had significant cognitive impairment, epilepsy, and sleep disturbance. Cognitive impairment in particular can delay diagnosis, because the child cannot communicate well with the caregiver. Moreover, changes in circadian cycle in the present case may have predisposed the patient to infection. Recent data have shown reciprocal connections between the central nervous system, sleep, and the immune system, as well as the importance of healthy sleep to maintain immune defenses (25). In addition, chronic sleep deprivation leads to sustained activation of the inflammatory response and increased risk of infectious disease, with major impairment of the antiviral response.

Several studies and meta-analyses have suggested asthma is unrelated to COVID-19 infection. In fact, the Th2 immunity in patients with asthma may be protective (26). Allergic sensitization seems to be inversely associated with ACE2 expression in the nasal epithelium, which is the main receptor through which SARS-CoV-2 enters the cell (26). However, another study showed that severe asthma can be a risk factor for increased mortality in hospitalized patients (27). In the present case, the patient's asthma was controlled, so it probably did not contribute to the severity of his condition.

Although the patient had produced a large amount of nAbs, it is not possible to determine whether these antibodies were responsible for his recovery or simply a consequence of intense, symptomatic disease. The attempt to use convalescent plasma to treat COVID-19 in clinical trials could shed light on this question; initial studies have presented positive data in this regard (28, 29). However, in one study including 228 patients with COVID-19 and pneumonia, some of the patients were treated with convalescent plasma, but the therapy showed no significant clinical benefit (30). Although it is not yet clear how much the nAbs contribute to recuperation from COVID-19, the use of pharmacological treatments has been further investigated.

When the patient was admitted to the hospital, azithromycin was thought to be effective against SARS-CoV-2, since it is commonly used to treat bacterial respiratory infections. Advances in research showed that the drug did not improve clinical outcomes over standard care (31).

Methylprednisolone was probably a main contributor to the patient's recovery, since several clinical trials have shown that administration of corticoids (dexamethasone, hydrocortisone, or methylprednisolone) is associated with lower mortality in critically ill patients with SARS-CoV-2, regardless of whether they are receiving mechanical ventilation (32).

The knowledge of COVID-19 infection in children is still sparse, and future studies must report the interaction between COVID-19 and particular syndromes. In particular, severe cases that are relevant to a specific subpopulation should be reported. To that end, in the present study, we presented the first documented case of a teenager with AS who had severe symptoms of COVID-19. The report might contribute to the understanding of COVID-19 and viral infection in patients with AS.

## Data Availability Statement

The raw data supporting the conclusions of this article will be made available by the authors, without undue reservation.

## Ethics Statement

The studies involving human participants were reviewed and approved by HMIMJ 223 4.042.665, HSL 4.045.959, ICB-USP 4.076.553. Written informed consent to participate in this study was provided by the participants' legal guardian/next of kin.

## Author Contributions

AL, DG, FO, DB, and MA designed the study. AL, CC, TB, and MA wrote the report. DG, FO, NC, and DB revised and added intellectual content. AL, AF, JB, RB, AB, and LM assisted with infection management of the patient and proofread the report. CC, TB, RB, GP, AS, LD, IA, DO, EB-J, LB-A, and LH provided the collection of data and biological sample from the patient. CC, TB, DO, RM, LT, and MH performed the experimental analyses. All authors contributed to the article and approved the submitted version.

## Conflict of Interest

MA and DO report grant from Fundação de Amparo à Pesquisa do Estado de São Paulo (FAPESP). RM reports fellowship from FAPESP. The remaining authors declare that the research was conducted in the absence of any commercial or financial relationships that could be construed as a potential conflict of interest.
